# Spatial segregation of mixed-sized counterions in dendritic polyelectrolytes

**DOI:** 10.1038/s41598-021-87448-9

**Published:** 2021-04-14

**Authors:** J. S. Kłos, J. Paturej

**Affiliations:** 1 Faculty of Physics, A. Mickiewicz University, Uniwersytetu Poznańskiego 2, 61-614 Poznań, Poland; 2 Leibniz Institute of Polymer Research Dresden e.V., Hohe Str. 6, 01069 Dresden, Germany; 3 Institute of Physics, University of Silesia, 75 Pułku Piechoty 1, 41-500 Chorzów, Poland

**Keywords:** Condensed-matter physics, Chemical physics

## Abstract

Langevin dynamics simulations are utilized to study the structure of a dendritic polyelectrolyte embedded in two component mixtures comprised of conventional (small) and bulky counterions. We vary two parameters that trigger conformational properties of the dendrimer: the reduced Bjerrum length, $\lambda _B^*$, which controls the strength of electrostatic interactions and the number fraction of the bulky counterions, $f_b$, which impacts on their steric repulsion. We find that the interplay between the electrostatic and the counterion excluded volume interactions affects the swelling behavior of the molecule. As compared to its neutral counterpart, for weak electrostatic couplings the charged dendrimer exists in swollen conformations whose size remains unaffected by $f_b$. For intermediate couplings, the absorption of counterions into the pervaded volume of the dendrimer starts to influence its conformation. Here, the swelling factor exhibits a maximum which can be shifted by increasing $f_b$. For strong electrostatic couplings the dendrimer deswells correspondingly to $f_b$. In this regime a spatial separation of the counterions into core–shell microstructures is observed. The core of the dendrimer cage is preferentially occupied by the conventional ions, whereas its periphery contains the bulky counterions.

## Introduction

Dendritic polyelectorolytes such as PAMAM (polyamidoamine) are extensively studied from the experimental and the theoretical point of view. Under physiological conditions only the terminal groups of PAMAMs bear positive charges. As the solution pH is lowered, the branching groups become protonated as well^1^. Ionization of the monomers provides a supply of counterions in solution, which has a tremendous effect on the structure and dynamics of the molecules. In this respect, absorption of both monovalent and divalent counterions^2^ into the pervaded volume of dendrimers and counterion condensation are of particular importance. These phenomena are challenging problems which are tackled through different methods including the Donnan, the Poisson-Boltzmann and the Manning–McGhee–von Hippel models^2,3^ as well as computer simulations^4–16^. Despite intensive studies of charged dendrimers in various environments and the role of counterions, still little is known about the effect of ion specificities on these polymers. In particular, an important factor is the size of counterions which is typically assumed to be either identical to the size of monomers or negligible^3,5–16^. Such an assumption is in contrast to several investigations which clearly demonstrated that the counterion size is a crucial control parameter. For instance, the behavior of polyelectrolyte gels in reaction to the size of counterions was examined using the virial expansion approximation^17^. It was recognized that the ion size has a substantial effect on the swelling ratio. Counterion specificity was also incorporated into the theory of phase transitions in polyelectrolyte gels^18^. It was demonstrated that small counterions generate volume collapse at low salt concentrations. Moreover, dissipative dynamics simulations were performed to study the conformational properties of polyelectrolyte chains accompanied by small and bulky counterions with various possibilities of the charged bead location^19^. Here, the main focus was on polymer collapse and formation of ionic microstructures. Molecular dynamics simulations were also employed to investigate the influence of the counterion steric effects on the structure of strongly charged polyelectrolytes in a dilute solution^20^. The authors reported formation of a dense globule in the presence of counterions of the size of monomers. For bulky counterions with the size much larger as compared to the monomer size, the polymers adopt extended conformations. Finally, for solutions containing a mixture of counterions of different size, formation of a core–shell globule takes place with the smaller counterions located within the globular core and the bulky counterions forming a shell on the globule surface. Some insights into the phenomena of ion selectivity between different counterions in the mixture was also gained for charged gels^21–24^, polyelectrolyte solutions^20,25^, the electric double layer around DNA^26^ and rodlike polyelectrolytes^27–30^. Last but not least, in our recent study we identified that the counterion size considerably affects conformations of charged dendrimers in a way that they swell to a larger extent in the presence of bulky counterions^31^.

Inspired by these studies, in this paper using Langevin dynamics simulations we continue to explore the structural properties of dendritic polyelectrolytes with the emphasis on the role of mixed-size counterions. We present the equilibrium properties of a charged dendrimer in a wide range of Coulomb interaction strength characterized by the reduced Bjerrum length, $\lambda ^*_B$. In addition, we focus on the impact of the counterion excluded volume induced by varied amounts of conventional and bulky counterions. For this purpose the composition of the ionic mixture is controlled by the number fraction of the bulky counterions, $f_b$. We find that both parameters $\lambda ^*_B$ and $f_b$ strongly affect conformations of charged dendrimers.

The paper is organized as follows. In “Model and method” section we outline the model and the simulation method. Our results are presented and discussed in “Results” section. We draw conclusions and remarks in “Summary” section.

## Model and method

### Model

We perform Langevin dynamics simulations of *G*4*S*4 charged dendrimer in a good, implicit solvent using the bead-spring model. In the above acronym, *G* represents dendrimer generation and *S* is the spacer length expressed in the number of bonds joining adjacent beads along a spacer chain. The tree-like structure of the molecule consists of the core of two bonded monomers and the trifunctional branching groups. The overall number of the monomers, *N*, and the branching groups (including the terminal groups), $N_{bg}$, in the dendrimer are given by

1 $$\begin{aligned} N=2+4S\left( 2^G-1 \right) =242, \end{aligned}$$

and

2 $$\begin{aligned} N_{bg}=2^{G+2}-2=62. \end{aligned}$$

**Figure 1:**
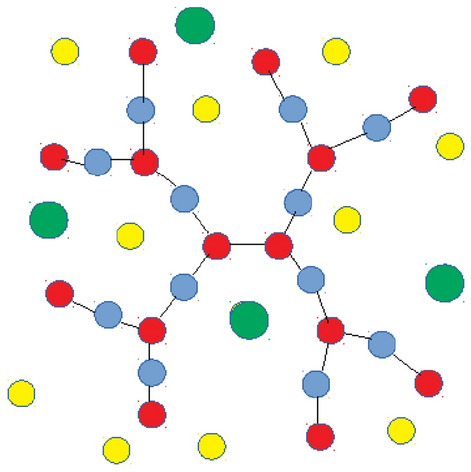
2D scheme of *G*2*S*2 charged dendrimer at low pH. The charged and the uncharged monomers are marked respectively by the red and the blue circles. The conventional and the bulky counterions are depicted by the yellow and the green circles.

We assign monovalent positive charges to the branching and the terminal groups so that the arrangement of charges corresponds to the architecture of PAMAM dendrimers at low pH^1^. In the following we interchangeably refer to the groups carrying charges as the charged monomers, the charged groups and the dendrimer ions. In order to maintain the overall system neutral, $N_{bg}$ monovalent negative counterions are included in the model. To study the dendrimer with mixed-size counterions, $N_{b}$ and $N_{c}=N_{bg}-N_{b}$ ions are considered as bulky and conventional, respectively, see Fig. 1. In the numerical scheme we incorporate three types of interactions. The excluded volume between pairs of particles of sizes, $\sigma _{\alpha }$, $\sigma _{\beta }$, with their centers separated by a distance *r*, accounted for via the Lennard–Jones (LJ) 12-6 truncated and shifted potential

3 $$\begin{aligned} U^{LJ}_{\alpha \beta }\left( r\right) = {\left\{ \begin{array}{ll} 4\epsilon \left[ \left( \frac{\sigma _{\alpha \beta }}{r}\right) ^{12}-\left( \frac{\sigma _{\alpha \beta }}{r}\right) ^{6} -\left( \frac{\sigma _{\alpha \beta }}{R^c_{\alpha \beta }}\right) ^{12} +\left( \frac{\sigma _{\alpha \beta }}{R^c_{\alpha \beta }}\right) ^{6} \right] , &\quad {\text {if }}\,r < R^{c}_{\alpha \beta } \\ 0, &\quad {\text{ otherwise }} \\ \end{array}\right. } \end{aligned}$$

where $\epsilon$ is the interaction strength, and $R^{c}_{\alpha \beta }=2^{1/6}\sigma _{\alpha \beta }$ is the cutoff radius. The indices $\alpha ,\beta$ = m, c, b, where m stands for monomers, c for conventional counterions and b for bulky ones, respectively. The choice of the cutoff results in purely repulsive LJ interactions mimicking good solvent conditions and, in Eq. (3), the mixing rule, $\sigma _{\alpha \beta }=\left( \sigma _{\alpha }+\sigma _{\beta }\right) /2$, is used. The bonds joining any two adjacent beads which are described by the Finite Extensible Non-linear Elastic potential (FENE)

4 $$\begin{aligned} U_{FENE}\left( r\right) = {\left\{ \begin{array}{ll} -0.5kR_0^2 \ln {\left( 1-\frac{r^2}{R_0^2} \right) }, &\quad {\text {if }}\,r < R_{0} \\ \infty , &\quad {\text{ otherwise }} \\ \end{array}\right. } \end{aligned}$$

where *k* is the spring constant and $R_0$ is the maximum extension of the bonds. The electrostatic interactions are introduced by pairwise Coulomb potential

5 $$\begin{aligned} \frac{U_{C}\left( r\right) }{k_BT}=\lambda _B\left( \varepsilon _r,T\right) \frac{z_{i}z_{j}}{r}, \end{aligned}$$

where $k_{B}$ denotes the Boltzmann constant, *T* the absolute temperature, $\lambda _B$ the Bjerrum length of the solvent, and *r* the distance between the centers of the *i*th and the *j*th particles with the charge valence $z_{i}$, $z_{j}=\pm 1$. With $\varepsilon _0$ standing for the electric permittivity of the vacuum, $\varepsilon _r$ for the relative permittivity of the solvent and *e* for elementary charge, the Bjerrum length is defined as $\lambda _B\left( \varepsilon _r,T\right) ={e^2}/({4\pi \varepsilon _0 \varepsilon _r k_BT})$. In particular, for water at room temperature ($T_r\approx 298$ K), $\varepsilon _{rw}\approx 81$, so that $\lambda _{B}\left( \varepsilon _{rw},T_r \right) \approx 7$ Å.

### Method

The LAMMPS molecular dynamics package^32^ was used to carry out the Langevin dynamics in the reduced LJ units. To set the unitless simulation parameters, the strength of the LJ potential, $\epsilon =1 k_BT_r$, and the monomer size, $\sigma _m=\lambda _{B}\left( \varepsilon _{rw},T_r \right)$, were taken as the real units of energy and length respectively. The unitless simulation input parameters (denoted with an asterisk) were chosen as $\epsilon ^*=1$ for the strength of the LJ potential, $\sigma _m^*$, $\sigma _c^*=1$ for the size of the monomers and the conventional counterions, and $m^*=1$ for the mass of the monomers and the counterions. For the FENE potential the parameters were $k^*=k\sigma _m^2/\epsilon =30$ and $R_0^*=R_0/\sigma _m=1.5$^33^. According to the formula for reduced elementary charge, $q^*=e/\left( 4\pi \varepsilon _0 \epsilon \sigma _m \right) ^{1/2}$, and for the reduced temperature, $T^*=k_BT/\epsilon$, we set these parameters to the values $q^*=9$ and $T^*=1$. Thus with the aforementioned energy unit, $\epsilon$, our simulations were carried out at the reduced temperature which corresponds to room temperature. Coulomb interactions were calculated using the Particle–Particle–Particle Mesh (PPPM) method with the error tolerance for force $10^{-4}$ and the real space cutoff radius $R^{c*}=10$^32^. The dumping parameter in the Langevin equation of motion was set to $\gamma ^*=\gamma / \tau =1$, where $\tau =\sigma _m\left( m/\epsilon \right) ^{1/2}$ is the LJ time unit and *m* is the assumed real unit of mass. For example, for $m\approx 30$ g/mol, $\tau \approx 2.4$ ps. The calculations were performed with a time step $\Delta t^*=\Delta t/\tau =0.005$. Note that the reduced Bjerrum length, $\lambda _{B}^*\left( \varepsilon _r,T_r\right) =\lambda _{B}\left( \varepsilon _r,T_r\right) /\sigma _m$, for a solvent at room temperature can be expressed as $\lambda _{B}^*\left( \varepsilon _r,T_r\right) ={\lambda _{B}^*\left( \varepsilon _{rw},T_r \right) }{\varepsilon _{rw}}/{\varepsilon _r}$, which with our unit of length yields $\lambda _{B}^*\left( \varepsilon _r,T_r\right) =\lambda _{B}^*={\varepsilon _{rw}}/{\varepsilon _r}$. This enabled us to vary $\lambda _{B}^*$ by changing the relative electric permittivity of the solvent, $\varepsilon _r$. According to Eq. (5), $\lambda _{B}^*$ is the parameter that determines Coulomb interaction energy between two monovalent charges at a distance $\sigma _m$ apart related to the thermal energy at room temperature. Furthermore, a crucial parameter is the density of particles in the simulation box. In the paper we present the results obtained in the counterion-only limit, which are valid for systems of well-dispersed dendritic polyelectrolytes and counterions at finite density fixed through periodic boundary conditions of a cubic simulation box of length $L^*=100$. The box size was large enough to prevent the dendrimer from intersecting with its periodic images, and allowed to perform simulations at experimentally accessible particle density: $\frac{N+N_{bg}}{L^{*3}}\sigma _{m}^{-3} \approx 3\cdot 10^{-4}\sigma _{m}^{-3}$. Typically, about $10^6$ equilibration integration steps were performed and followed by $10^7$ integration steps of the production runs. During the production runs we collected the data on the scale up to hundreds of the longest relaxation times $\tau _D$, where $\tau _D$ is defined as time needed for dendrimer to diffuse its own size. All G4 dendrimers considered in the current study had $\tau _D<1000$ $\tau$. The snapshots of the monomers’ positions used in the analysis of the structural properties were saved every 1000 time steps. Throughout the paper our focus is on the conformational properties of the dendritic polyelectrolyte accompanied by binary mixtures of the conventional and the bulky counterions. The size of the bulky ions was $\sigma _b^*=\sigma _b/\sigma _m=2$, 3, and their number fraction was set to $f_b=N_{b}/N_{bg}=0, 0.16, 0.32, 0.48, 0.64, 0.8, 1$. The reduced Bjerrum length, $\lambda _{B}^*$, was varied between 0.125 and 16, covering the extremes of both weak and strong electrostatic interactions. In the following we refer to the counterions with a diameter $\sigma _c^*=1$ as the conventional (counter)ions and with a diameter $\sigma _b^*=2$, 3 as the bulky ones.

The simulation snapshots were rendered using the visual molecular dynamics (VMD)^34^. All plots were prepared using XMGRACE^35^.

## Results

### Spatial distribution of counterions

**Figure 2:**
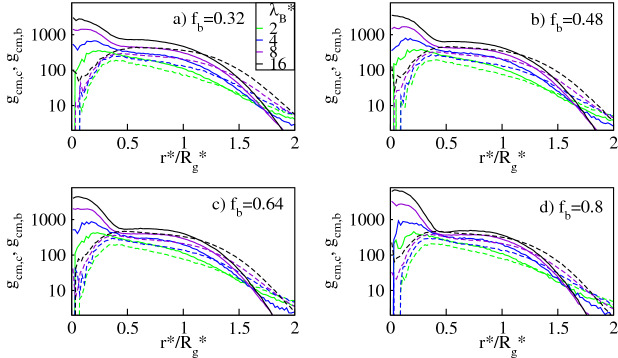
Log-lin plot of the pair correlation function for the conventional counterions, $g_{cm,c}$ (solid lines), and the bulky counterions with a diameter $\sigma ^*_b=2$, $g_{cm,b}$ (dashed lines), calculated around the dendrimer’s center-of-mass at various Bjerrum lengths, $\lambda _B^*$, for fixed number fractions of the bulky counterions, $f_b$, as indicated. In all the panels the maximum error bar is less than $5\%$.

In Fig. 2 we display the pair correlation function $g_{cm,c}$ and $g_{cm,b}$ for the conventional and the bulky counterions respectively, calculated around the dendrimer’s center-of-mass. The profiles are presented as functions of the radial distance, $r^*/R_g^*$, from the dendrimer’s center-of-mass rescaled by the dendrimer’s radius of gyration, $R_g^*$, for different Bjerrum lengths, $\lambda _B^*$, and a given number fraction of the bulky counterions, $f_b$. The data demonstrate that both types of ionic species accumulate at distances $r^*/R^*_g\lessapprox 2$ from the dendrimer’s core. The absorption effect is enhanced with increasing $\lambda _B^*$, which is manifested by an increase in the pair correlation function in this volume. The latter effect is attributed to stronger Coulomb attraction between the counterions and the dendrimer ions. Our finding confirms the previous numerical results, which however, were solely restricted to the case of conventional counterions^5–16^. As shown in Fig. 2, the pair correlation functions reveal a strong dependence on the ionic type. For $\lambda _B^*\gtrapprox 4$ the $g_{cm,c}$-profiles peak in a neighborhood of the center of mass, flatten in the molecule’s domain, and drop on its periphery. The $g_{cm,b}$-profiles exhibit a broad maximum in the domain and decrease near the center and on the periphery. Thus for sufficiently large $\lambda _B^*$-values, the dendrimer’s core is rich in the conventional counterions due to their weak excluded volume and Coulomb attraction with the charged monomers. On the other hand, on the periphery of the dendrimer an opposite tendency occurs due to more pronounced steric repulsion of the bulky ions with the dense core of the molecule, see Fig. 14. For the bulky counterions stronger excluded volume interactions prevent them from occupying the dendrimer’s core, which results in their accumulation at a large distance from it. Note that a similar preference of the counterions to spatially separate was also observed for linear polyelectrolytes^20^. On this ground we conclude that an ionic microseparation caused by the presence of mixed-size counterions is a universal phenomenon which occurs for polyelectrolytes with various molecular topologies.

**Figure 3:**
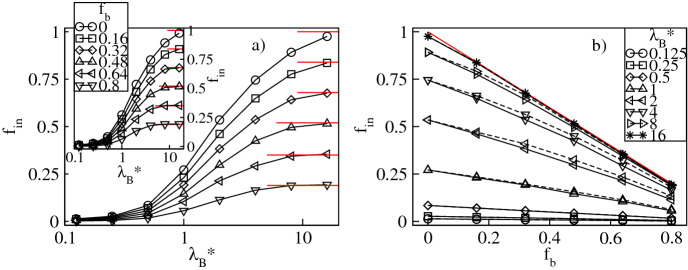
Fraction, $f_{in}$, of the conventional counterions absorbed into the dendrimer versus the Bjerrum length, $\lambda _B^*$, plotted for a fixed number fraction, $f_{b}$, of the bulky counterions with a diameter $\sigma ^*_b=2$ ($\sigma ^*_b=3$ in the inset) on the lin-log scale (**a**), and versus $f_{b}$ for fixed $\lambda _B^*$ on the lin–lin scale (**b**). In (**b**) the solid (dashed) lines represent the data for $\sigma ^*_b=2$ ($\sigma ^*_b=3$). The red lines indicate the number fraction, $1-f_b$, of the conventional counterions. Here and in the remaining plots the error bars for all the data points are smaller than the symbol size.

**Figure 4:**
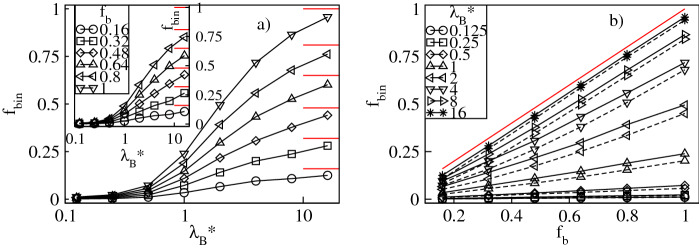
Fraction, $f_{bin}$, of the bulky counterions absorbed into the dendrimer versus the Bjerrum length, $\lambda _B^*$, plotted for a fixed number fraction, $f_{b}$, of the bulky counterions with a diameter $\sigma ^*_b=2$ ($\sigma ^*_b=3$ in the inset) on the lin-log scale (**a**), and versus $f_{b}$ for fixed $\lambda _B^*$ on the lin–lin scale (**b**). In (**b**) the solid (dashed) lines represent the data for $\sigma ^*_b=2$ ($\sigma ^*_b=3$). The red lines indicate the number fraction, $f_b$, of the bulky counterions.

**Figure 5:**
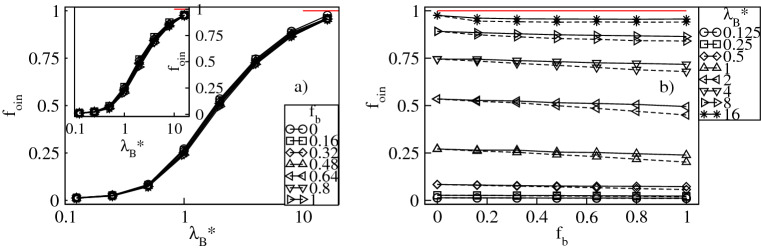
Overall fraction, $f_{oin}$, of the counterions absorbed into the dendrimer versus the Bjerrum length, $\lambda _B^*$, plotted for a fixed number fraction, $f_{b}$, of the bulky counterions with a diameter $\sigma ^*_b=2$ ($\sigma ^*_b=3$ in the inset) on the lin-log scale (**a**), and versus $f_{b}$ at fixed $\lambda _B^*$ on the lin–lin scale (**b**). In (**b**) the solid (dashed) lines represent the data for $\sigma ^*_b=2$ ($\sigma ^*_b=3$). The red lines indicate $f_{oin}=1$.

To analyze the uptake of the counterions quantitatively, in Figs. 3a and 4a we display respectively the fractions of the absorbed conventional, $f_{in}=N_{in}/N_{bg}$, and bulky, $f_{bin}=N_{bin}/N_{bg}$, counterions versus $\lambda _B^*$ at constant values of $f_b$. The fractions are calculated with respect to the overall number of the counterions, $N_{bg}$. Furthermore, $N_{in}$ and $N_{bin}$ denote the mean numbers of the conventional and the bulky counterions at distances less than $2R_g^*$ from the dendrimer’s center-of-mass. Our criterion for absorption is based on that both the radial densities of the monomers and the counterions are nearly zero at $r^*/R_g^*\approx 2$, see Figs. 2 and 14. Figure 3a demonstrates that $f_{in}$ increases monotonically from nearly zero up to $1-f_b$ with increasing $\lambda ^*_B$. Thus, for strong Coulomb interactions all the conventional counterions present in the mixture penetrate the dendrimer, whereas for weak electrostatic couplings absorption of these ions is minor. A monotonic increase is also found for $f_{bin}$, see Fig. 4a. In particular, no absorption of the bulky counterions occurs for weak electrostatic interactions, whereas in the limit of large $\lambda _B^*$-values it is almost complete since $f_{bin}\approx f_b$. Therefore, an uptake of the counterions of both types takes place and is enhanced accordingly to $f_b$ as $\lambda _B^*$ is increased. In Figs. 3b and 4b we display both fractions as functions of $f_b$ at constant $\lambda _B^*$. Our data indicate that increasing $f_b$ results in a monotonic reduction of $f_{in}$ from the maximum at $f_b=0$ corresponding to the dendrimer with only conventional counterions. In turn, $f_{bin}$ is found to increase monotonically up to the maximum at $f_{b}=1$ corresponding to the polyelectrolyte accompanied by only the bulky counterions. Note that absorption of the bulky counterions with a diameter $\sigma ^*_b=3$ is slightly weaker than of those with $\sigma ^*_b=2$. More interestingly, we observe that the variation of both fractions is almost linear with $f_b$ and steeper at larger $\lambda _B^*$-values. Last but not least, we analyze the overall fraction of the absorbed counterions, $f_{oin}=f_{in}+f_{bin}$. Figure 5a demonstrates $f_{oin}$ versus $\lambda _B^*$ at constant $f_b$. In accordance with the behavior of its components $f_{oin}$ is a monotonically increasing function of $\lambda _B^*$ from nearly zero up to one. Note that the $f_{oin}$-profiles are only weakly affected by the $f_b$-values and tend to collapse onto a single curve. This observation is also confirmed in Fig. 5b, which demonstrates that $f_{oin}$ is almost a constant function of $f_{b}$ at fixed $\lambda _B^*$. Thus, the absorption process of the counterions is basically determined by Coulomb interactions as long as no distinction between the ions is made. Variations in the composition of the ionic mixture at constant Bjerrum length have only a weak impact on the overall amount of the absorbed counterions.

### Condensed and delocalized counterions

**Figure 6:**
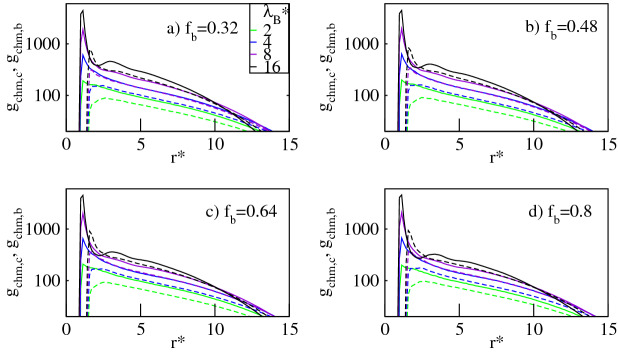
Log-lin plot of the pair correlation function, $g_{chm,c}$ (solid lines), $g_{chm,b}$ (dashed lines), for the conventional and the bulky counterions with a diameter $\sigma ^*_b=2$ respectively, calculated around the charged groups of the dendrimer at various Bjerrum lengths, $\lambda _B^*$, for fixed number fractions of the bulky counterions, $f_b$, as indicated. In all the panels the maximum error bar is less than $5\%$.

As demonstated in a number of studies a fraction of the absorbed counterions condense on the charged groups of dendritic polyelectrolytes^8–11,14^. Our data indicate that this is also the case for dendrimers with a mixture of conventional and bulky counterions. This tendency is shown in Fig. 6, in which we display the charged monomer-conventional counterion, $g_{chm,c}$, and the charged monomer-bulky counterion, $g_{chm,b}$, pair correlation functions, respectively. Pronounced maxima of the profiles at $r^*_{\alpha }\approx \left( 1+\sigma _{\alpha }^*\right) /2$ ($\alpha$ =  c, b) indicate that the counterions of both types condense on the dendrimer ions. This phenomenon is enhanced for larger $\lambda _B^*$ values due to stronger Coulomb attraction between the oppositely charged species, which is manifested by an increase in the height of the maxima. Obviously, the fact that $r^*_{b}>r^*_{c}$ is related to the difference in the counterion size. Note that at given $f_b$ and $\lambda _B^*$ the maxima of the $g_{chm,c}$-profiles are sharper as compared with these of the $g_{chm,b}$-profiles, which indicates that the conventional counterions are more likely to penetrate the vicinity of the dendrimer ions. The small size of the conventional ions enables them to approach the charged groups of the dendrimer more efficiently and to be more strongly electrostatically bound with them. On the other hand, the bulky counterions are prevented from gathering in close proximity of the charged monomers due to their stronger excluded volume.

**Figure 7:**
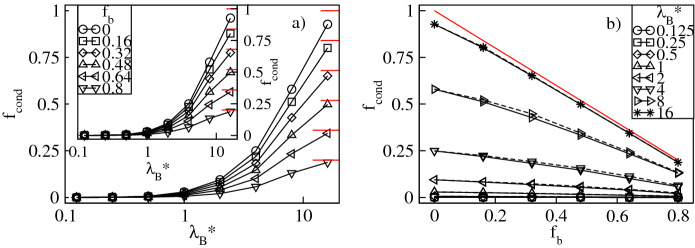
Fraction, $f_{cond}$, of the condensed conventional counterions versus the Bjerrum length, $\lambda _B^*$, plotted for a fixed number fraction, $f_{b}$, of the bulky counterions with a diameter $\sigma ^*_b=2$ ($\sigma ^*_b=3$ in the inset) on the lin-log scale (**a**), and versus $f_{b}$ at fixed values of $\lambda _B^*$ on the lin–lin scale (**b**). In (**b**) the solid (dashed) lines represent the data for $\sigma ^*_b=2$ ($\sigma ^*_b=3$). The red lines indicate $1-f_b$.

**Figure 8:**
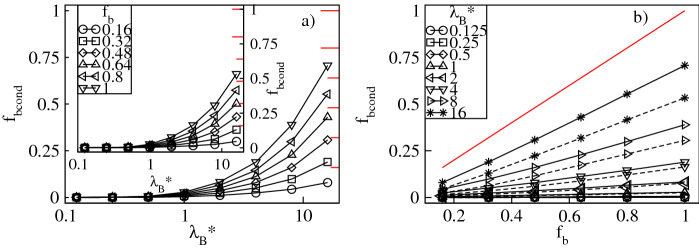
Fraction, $f_{bcond}$, of the condensed bulky counterions versus the Bjerrum length, $\lambda _B^*$, plotted for a fixed number fraction, $f_{b}$, of the bulky counterions with a diameter $\sigma ^*_b=2$ ($\sigma ^*_b=3$ in the inset) on the lin-log scale (**a**), and versus $f_{b}$ at fixed values of $\lambda _B^*$ on the lin–lin scale (**b**). In (**b**) the solid (dashed) lines represent the data for $\sigma ^*_b=2$ ($\sigma ^*_b=3$). The red lines indicate $f_b$.

**Figure 9:**
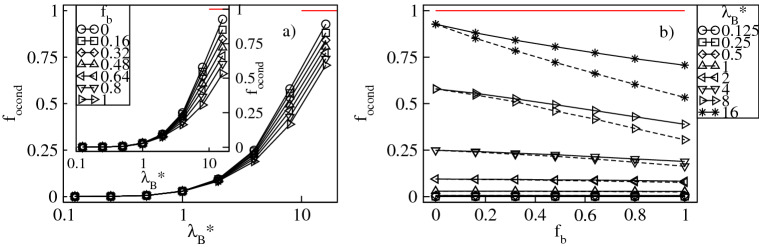
Overall fraction, $f_{ocond}$, of the condensed counterions versus the Bjerrum length, $\lambda _B^*$, plotted for a fixed number fraction, $f_{b}$, of the bulky counterions with a diameter $\sigma ^*_b=2$ ($\sigma ^*_b=3$ in the inset) on the lin-log scale (**a**), and versus $f_{b}$ at fixed values of $\lambda _B^*$ on the lin–lin scale (**b**). In (**b**) the solid (dashed) lines represent the data for $\sigma ^*_b=2$ ($\sigma ^*_b=3$). The red lines indicate $f_{ocond}=1$.

To quantify the degree of counterion condensation, in Figs. 7a and 8a we plot respectively the fractions, $f_{cond}=N_{cond}/N_{bg}$ and $f_{bcond}=N_{bcond}/N_{bg}$, of the mean number of the condensed conventional and bulky counterions with respect to their overall number in the mixture as functions of $\lambda _B^*$ at constant $f_b$. Our criterion for condensation is based on the distance between the counterions and the dendrimer ions. A counterion is considered condensed if its distance from at least one charged monomer is less than $\left( 2+\sigma _{\alpha }^*\right) /2$, where $\alpha =$ c,b. In accordance with the previous investigations of charged dendrimers with solely conventional counterions^8–11,14^, we find a monotonic increase in $f_{cond}$ and $f_{bcond}$ with $\lambda _B^*$. Specifically, irrespective of the $f_b$-values, for $\lambda _B^* \lessapprox 1$ the thermal energy dominates over Coulomb attraction between the opposite charges and condensation cannot be achieved, i.e, $f_{cond}$ and $f_{bcond}$ are nearly zero. For $\lambda _B^* >1$ the electrostatic attraction dominates and promotes counterion condensation. In this regime $f_b$ is already a selective parameter which causes a split of both fractions. In particular, for small $f_b$-values $f_{cond}$ increases abruptly, whereas the increase in $f_{bcond}$ is minor. An opposite tendency is observed for large $f_b$-values. Note that in the limit of strong Coulomb couplings almost all the conventional counterions present in the mixture are condensed, whereas condensation of the bulky ions still remains incomplete. The latter observation is attributed to weaker electrostatic binding of the bulky counterions in contact with the charged monomers and strong steric repulsions that prevent such contacts. The effect of variation of $f_b$ on condensation of the counterions of both types at constant $\lambda _B^*$ is presented in Figs. 7b and 8b. Here, both ionic fractions follow the behavior previously discussed for the fractions of the absorbed counterions. Namely, $f_{cond}$ decreases from the maximum at $f_b=0$ and $f_{bcond}$ increases up to the maximum at $f_b=1$ almost linearly. As compared with the intermediate cases ($0<f_b<1$), the system containing only the conventional counterions ($f_b=0$) is characterized by the largest fraction of the condensed conventional counterions. Similarly, the fraction of the condensed bulky counterions is the largest in the other extreme of the dendrimer accompanied by the bulky ions only ($f_b=1$). Furthermore, the change in the number of both condensed components is more abrupt at larger $\lambda _B^*$-values as well. Note that the effect of $\sigma ^*_b$ on condensation of the conventional counterions is rather minor, whereas for the bulky ones clearly visible. In the latter case, at given $f_b$ a drop in $f_{bcond}$ is observed for the bulky counterions with a diameter $\sigma ^*_b=3$ since these ions are more weakly bound with the charged groups. This also results in a smaller slope of the $f_{bcond}$-profiles as functions of $f_b$. To complement our analysis of condensation in Fig. 9 we display the overall fraction of the condensed counterions, $f_{ocond}=f_{cond}+f_{bcond}$. For $\lambda _B^*\lessapprox 1$ the overall amount of the condensed counterions is negligible, whereas in the other regime of strong Coulomb interactions the overall condensation effect is significantly enhanced, see Fig. 9a. In particular, for $\lambda _B^*\gtrapprox 4$ the $f_{ocond}$-profiles increase abruptly according to $f_b$. Note that the most abrupt increase of condensation takes place at $f_b=0$, i.e., for the dendrimer with only the conventional counterions. The slowest growth occurs in the other extreme of the polyelectrolyte accompanied by the bulky ions only. Figure 9b demonstrates $f_{ocond}$ as a function of $f_b$ at constant $\lambda _B^*$. It confirms that counterion condensation does not take place in the limit of weak electrostatic interactions. Above the crossover at $\lambda _B^*\approx 1$ a nearly linear decrease in $f_{ocond}$ is observed, which is steeper for the bulky counterions with a diameter $\sigma ^*_b=3$. Thus, replacing the conventional counterions with the bulky ones weakens the overall condensation effect. The maximum in the overall fraction of the condensed counterions is obtained for the dendrimer with solely the conventional counterions and decays with increasing the amount of the bulky ions.

**Figure 10:**
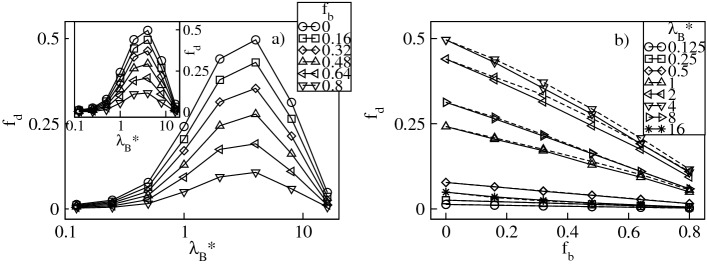
Fraction, $f_{d}$, of the delocalized conventional counterions versus the Bjerrum length, $\lambda _B^*$, plotted for a fixed number fraction, $f_{b}$, of the bulky counterions with a diameter $\sigma ^*_b=2$ ($\sigma ^*_b=3$ in the inset) on the lin-log scale (**a**), and versus $f_{b}$ at fixed values of $\lambda _B^*$ on the lin–lin scale (**b**). In (**b**) the solid (dashed) lines represent the data for $\sigma ^*_b=2$ ($\sigma ^*_b=3$).

**Figure 11:**
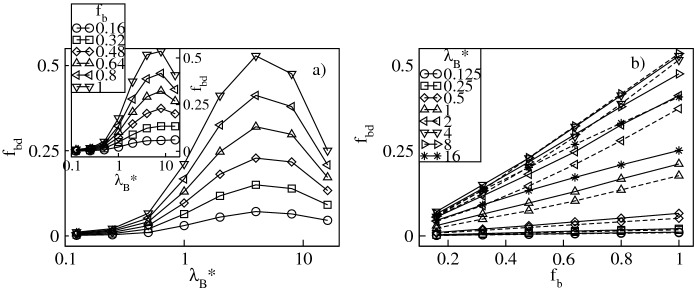
Fraction, $f_{bd}$, of the delocalized bulky counterions versus the Bjerrum length, $\lambda _B^*$, plotted for a fixed number fraction, $f_{b}$, of the bulky counterions with a diameter $\sigma ^*_b=2$ ($\sigma ^*_b=3$ in the inset) on the lin-log scale (**a**), and versus $f_{b}$ at fixed values of $\lambda _B^*$ on the lin–lin scale (**b**). In (**b**) the solid (dashed) lines represent the data for $\sigma ^*_b=2$ ($\sigma ^*_b=3$).

**Figure 12:**
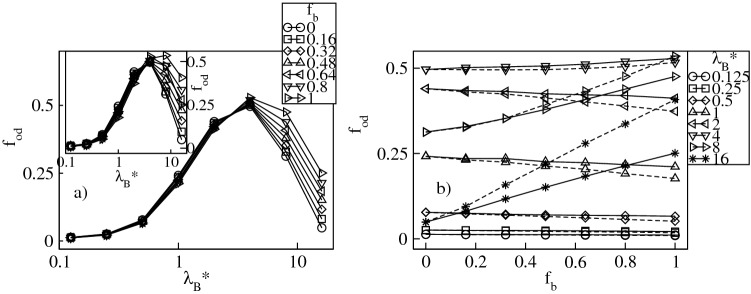
Overall fraction, $f_{od}$, of the delocalized counterions versus the Bjerrum length, $\lambda _B^*$, plotted for a fixed number fraction, $f_{b}$, of the bulky counterions with a diameter $\sigma ^*_b=2$ ($\sigma ^*_b=3$ in the inset) on the lin-log scale (**a**), and versus $f_{b}$ at fixed values of $\lambda _B^*$ on the lin–lin scale (**b**). In (**b**) the solid (dashed) lines represent the data for $\sigma ^*_b=2$ ($\sigma ^*_b=3$).

Besides condensed counterions, inside the dendrimer there are also delocalized ions which freely penetrate the molecule and exert the osmotic pressure^9^. Figure 10a displays the fraction of the delocalized conventional counterions, $f_d=f_{in}-f_{cond}$, whereas in Fig. 11a we show the fraction of the delocalized bulky counterions, $f_{bd}=f_{bin}-f_{bcond}$. Both plots are presented as functions of $\lambda _B^*$ at constant $f_b$. Our data indicate that both ionic fractions are non-monotonic functions of $\lambda _B^*$ and exhibit a broad maximum. Before the maximum it is absorption of the counterions of both ionic types that increases more rapidly with $\lambda _B^*$ than their condensation. For strong Coulomb couplings an opposite tendency takes place, i.e., counterion condensation is dominant and results in a decrease in $f_d$ and $f_{bd}$. Note that the $f_d$-maximum is the highest in the presence of solely the conventional ions and flattens with increasing $f_b$. On the other hand, the $f_{bd}$-fraction is the largest in the presence of solely the bulky ions and flattens as $f_b$ is decreased. Moreover, at $\lambda _B^*\approx 16$ there are no delocalized conventional counterions due to their complete condensation, whereas the amount of the delocalized bulky ions is still finite. In Figs. 10b and 11b we display both ionic fractions versus $f_b$ at constant $\lambda _B^*$. A monotonic decrease in $f_d$ and a monotonic increase in $f_{bd}$ is observed. Deviations from linear behavior of the corresponding ionic fractions are weak though more noticeable for systems with the bulky ions with a diameter $\sigma ^*_b=3$. For both ionic fractions we do not find a clear, monotonic dependence of the slopes on $\lambda _B^*$. Finally, in Fig. 12a we display the overall fraction of the delocalized counterions, $f_{od}=f_{d}+f_{bd}$, versus $\lambda _B^*$. Similar to its components, $f_{od}$ is a non-monotonic function of $\lambda ^*_B$ exhibiting a broad maximum around $\lambda _B^* \approx 4$ which corresponds to the crossover between the two regimes of counterion absorption dominating over counterion condensation and vice versa. Note that in line with the $f_{ocond}$-profiles, the effect of varying $f_b$ becomes noticeable above the crossover. The latter observation is also confirmed in Fig. 12b which demonstrates $f_{od}$ as a function of $f_b$. Namely, for $\lambda _B^* \lessapprox 4$ only a weak dependence of $f_{ocond}$ on $f_b$ is observed, whereas above the crossover an almost linear, sharp increase in the $f_{od}$-fraction takes place that is more pronounced for $\sigma ^*_b=3$. The latter conclusion is another manifestation of the fact that the larger bulky counterions are more weakly bound with the charged groups as compared to the counterions with a smaller diameter.

### Radius of gyration

**Figure 13:**
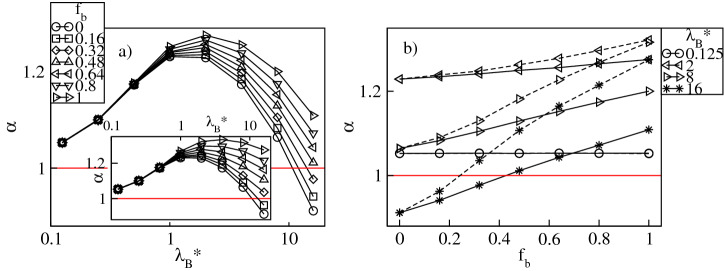
Swelling factor, $\alpha$, versus the Bjerrum length, $\lambda _B^*$, plotted for a fixed number fraction, $f_{b}$, of the bulky counterions with a diameter $\sigma ^*_b=2$ ($\sigma ^*_b=3$ in the inset) on the lin-log scale (**a**), and versus $f_{b}$ at fixed values of $\lambda _B^*$ on the lin–lin scale (**b**). In (**b**) the solid (dashed) lines represent the data for $\sigma ^*_b=2$ ($\sigma ^*_b=3$). The red lines indicate $\alpha =1$.

**Figure 14:**
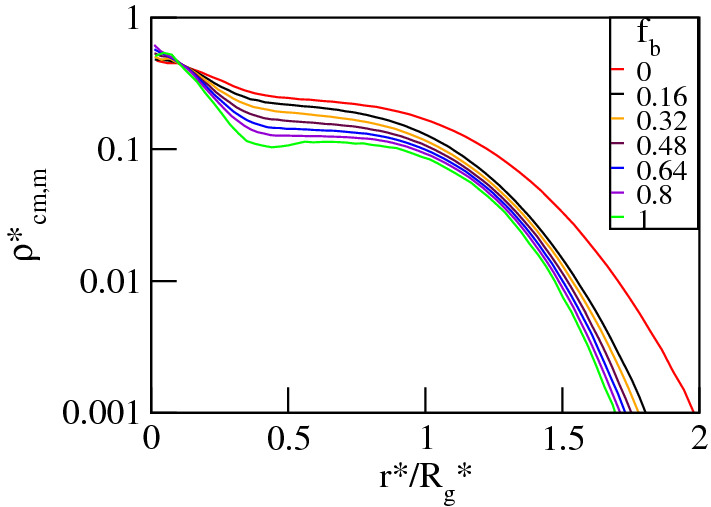
Log-lin plot of the radial density profiles, $\rho _{cm,m}^*$, of the monomers around the center-of-mass of the dendrimer plotted for various fractions, $f_b$, of the bulky counterions with a diameter $\sigma ^*_b=2$ at $\lambda _B^*=16$. The maximum error bar for all the data sets is less than $5\%$.

**Figure 15:**
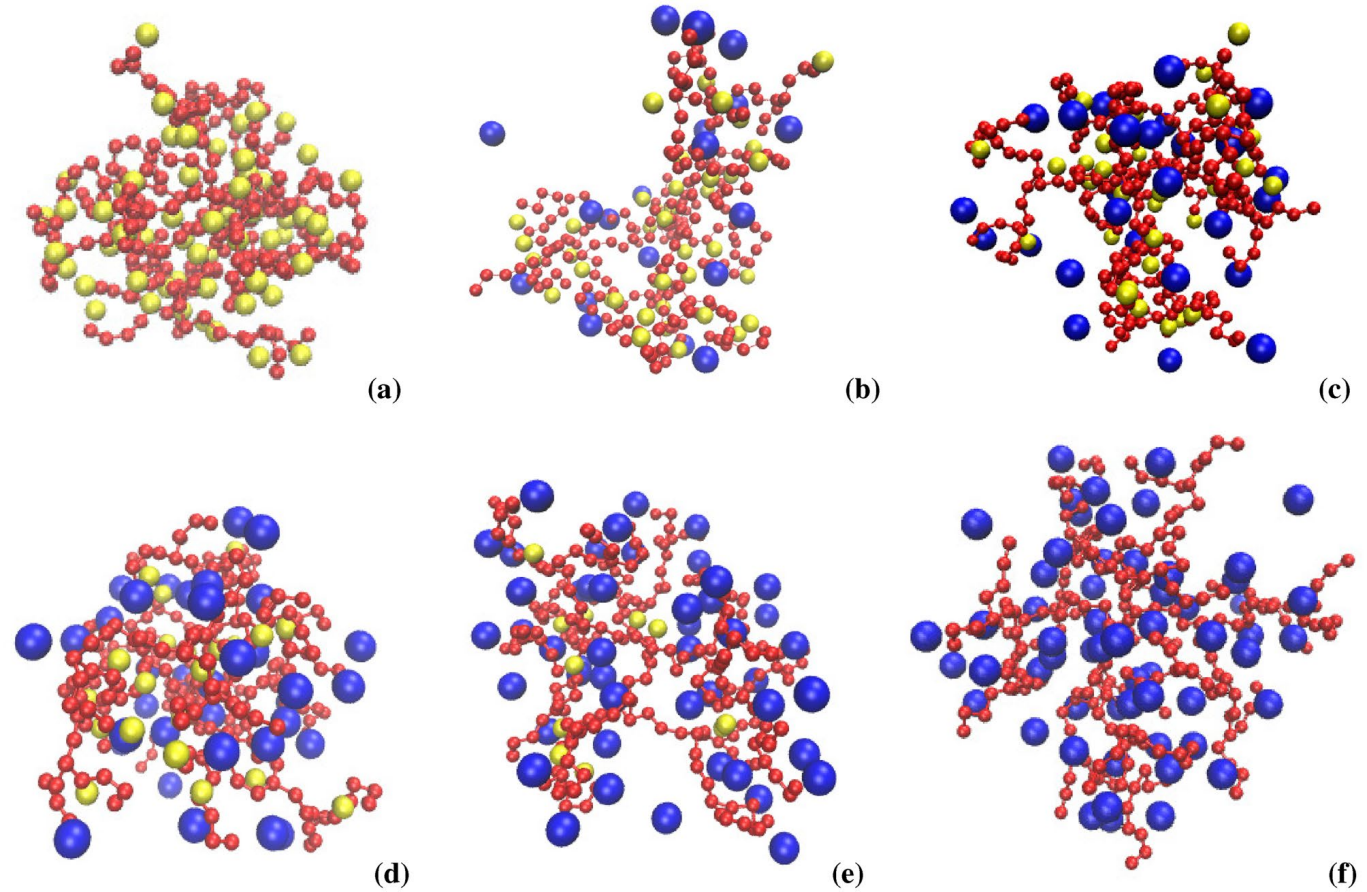
Snapshots of conformations of the dendrimer with mixed-size counterions at $\lambda _B^*=16$ for fixed fractions of the bulky counterions with a diameter $\sigma ^*_b=2$: $f_b=0$ (**a**), $f_b=0.32$ (**b**), $f_b=0.48$ (**c**), $f_b=0.64$ (**d**), $f_b=0.8$ (**e**), $f_b=1$ (**f**). The monomers (and the conventional/bulky counterions) are displayed by red (yellow/blue) spheres.

In Fig. 13 we display the swelling factor of the dendritic polyelectrolyte, $\alpha = R_g^*/R_{g0}^*$, defined as the ratio between the radius of gyration of the charged dendrimer and its neutral analogue versus $\lambda _{B}^*$ and $f_b$. The data indicate that as a function of $\lambda _{B}^*$ the dendrimer’s size changes non-monotonically in the whole range of the $f_b$-values, see Fig. 13a. Increasing $\lambda _{B}^*$ causes swelling of the polymer up to a broad maximum followed by deswelling observed for strong Coulomb couplings. The dependence of $\alpha$ is qualitatively similar to the outcomes of the previous simulations of dendritic polyelectrolytes carried out for solely conventional ions^8,10,11,14^. The equilibrium size of the dendrimer results from a balance between the elasticity of the molecule and intramolecular interactions of different origin. Namely, for $\lambda _{B}^*\lessapprox 1$ absorption of the counterions is minor and $\alpha$ is unaffected by both $f_b$ and $\sigma ^*_b$. In this regime swelling is mostly due to Coulomb repulsion between the charged groups of the dendrimer. Ionic specificity becomes essential for $\lambda _{B}^*\gtrapprox 1$ where penetration of the dendrimer’s pervaded volume by the counterions is significant and leads to a split in $\alpha$ depending on the value of $f_b$. The $\alpha$-profiles exhibit maxima at $1\lessapprox \lambda _{B}^*\lessapprox 4$ which correspond with the maximum of the overall fraction of the delocalized counterions, $f_{od}$. Here, the high osmotic pressure due to the delocalized counterions of both types and the unscreened electrostatic repulsion between the dendrimer ions lead to the pronounced swelling effect^8,9^. Furthermore, the maximum of $\alpha$ increases with varying $f_b$ from zero to one. Given the fact that in the considered $\lambda _{B}^*$-range the drop in the fractions $f_{in}$ and $f_d$ is accompanied by the increase in the fractions $f_{bin}$ and $f_{bd}$ such that their sums, $f_{oin}$ and $f_{od}$, only weakly depend on $f_b$, the $f_b$-induced enhancement of swelling is attributed to a larger contribution of the excluded volume interactions to the osmotic pressure caused by the absorbed bulky counterions. Subsequently, at $\lambda _{B}^*\gtrapprox 4$ counterion condensation gradually becomes dominant. It screens the intramolecular Coulomb repulsion and suppresses the impact of the delocalized ions, which effectively results in polymer deswelling. Note that for the dendrimer accompanied by ionic mixtures with $f_b\lessapprox 0.32$ there exist $\lambda _{B}^*$-values at which the molecule recovers its size in the neutral state, and a further increase in $\lambda _{B}^*$ promotes its collapse. In the latter case the radius of gyration is mostly determined by excluded volume repulsions and multiple attractions between ion pairs formed by the condensed counterions and the charged groups^10,36,37^. For counterion mixtures with $f_b\gtrapprox 0.32$ a reduction of the dendrimer size occurs as well, though the molecule still remains swollen. The effect of $f_b$ on $\alpha$ is also displayed in Fig. 13b. This figure clearly shows that $\alpha$ increases monotonically with $f_b$ at constant $\lambda _{B}^*\gtrapprox 1$. In particular, at $\lambda _{B}^*\approx 16$ increasing $f_b$ from zero to one leads to a structural transition of the dendrimer from collapsed to swollen conformations. This kind of transiton is due to weaker counterion condensation which reduces the effect of the multiple attraction promoting shrinking and enhances the impact of the aforementioned sources of intramolecular repulsions. The conformational changes of the dendrimer are also reflected by the spatial distribution of the monomers shown in Fig. 14. Here, we display the density profiles of the monomers, $\rho _{cm,m}^*$, as functions of the rescaled radial distance from the dendrimer’s center-of-mass at $\lambda _{B}^*=16$. The collapse of the polymer with decreasing $f_b$ is signaled by an increase in $\rho _{cm,m}^*$, especially within the dendrimer’s pervaded volume. Moreover, our simulations confirm that the dendrimer exists in dense-core conformations^8^. The monomer densities are characterized by a sharp peak in a neighborhood of the center-of-mass, a broad plateau in the polymer’s domain and a drop on the periphery. Note that the shape of the $g_{cm,c}$-profiles to some extent follows the density profiles of the monomers. This is another manifestation of pronounced condensation of the conventional ions which predominantly occupy a close proximity of the charged groups at large Bjerrum lengths. From this we also conclude that the emergence of the ionic core–shell structures inside the molecule is mostly driven by selective condensation of the counterions. We visualize counterion absorption into the dendrimer in Fig. 15 by displaying a series of snapshots from our MD simulations at $\lambda _B^*=16$ for various values of $f_b$

.

## Summary

In this work using Langevin dynamics simulations we studied *G*4*S*4 dendritic polyelectrolyte with two component mixtures of conventional and bulky counterions. The main focus of our investigation was on the conformational behavior of the molecule modified by the reduced Bjerrum length, $\lambda _B^*$, and the number fraction of the bulky counterions, $f_b$. Our simulations indicate that irrespective of the amount of the bulky counterions in the ionic mixture the dendrimer swells non-monotonically with increasing $\lambda _B^*$. At small Bjerrum lengths, $\lambda _{B}^* \lessapprox 1$, the counterions are distributed uniformly in solution and their density within the dendrimer’s pervaded volume is negligible. As a consequence, the radius of gyration of the polyelectrolyte exceeds that of the neutral dendrimer due to Coulomb repulsion between the like-charged monomers and remains unaffected by the ions. At intermediate Bjerrum lengths, $1\lessapprox \lambda _B^*\lessapprox 4$, the electrostatic attraction between the counterions and the dendrimer ions promotes absorption of the former into the dendrimer’s pervaded volume and their condensation. Inside the dendrimer the densities of the counterions of both types become significant and $f_b$ starts to affect the molecule’s conformations. In this regime, for a given value of $f_b$, the $\lambda _B^*$-dependent radius of gyration of the dendrimer exhibits a broad maximum, which corresponds with the maximum of the overall fraction of the absorbed, delocalized counterions. Here, swelling is primarily attributed to the high osmotic pressure exerted by the delocalized counterions on the polymer and unscreened intramolecular Coulomb repulsion between the dendrimer ions. Moreover, swelling increases monotonically with $f_b$ due to the enhanced contribution of the excluded volume interactions between the particles penetrating the dendrimer’s interior to the osmotic pressure. Subsequently, for large Bjerrum lengths, $\lambda _B^*\gtrapprox 4$, condensation of the conventional and the bulky counterions is significant and determines the conformational behavior of the polymer. In this regime deswelling and eventually collapse of the dendrimer into a globule-like state takes place, the latter being attributed to formation of condensed-ion/charged-monomer pairs possessing a net attraction. Increasing $f_b$ weakens counterion condensation as well as the impact of the intramolecular attractive forces, and a departure of the polyelectrolyte from collapsed to swollen conformations is observed. Our simulations show that the absorbed counterions tend to separate into core–shell microstructures. The core of the dendrimer is rich in the conventional ions, whereas the volume around it contains the bulky ions. A similar observation was made for strongly charged linear chains^20^. Our study indicates that conformations of highly branched macromolecules tend to reveal an ionic separation facilitated by selective condensation of conventional and bulky counterions. It seems likely that this phenomenon is generic in nature and does not depend on specific arrangements of branching nodes. Thus a broad range of polymeric architectures including dendrimers of higher generations and with different spacer lengths, dendronized, pom-pom-shaped, star-burst and bottlebrush polymers^38–40^ as well as microgels^41^ are expected to demonstrate similar microseparation effects. These systems are clearly a motivation for further studies. Another interesting subject for the future work is the influence of pH conditions and charge arrangements on the counterion segregation.
